# Jasmonic Acid Modulates the Physio-Biochemical Attributes, Antioxidant Enzyme Activity, and Gene Expression in *Glycine max* under Nickel Toxicity

**DOI:** 10.3389/fpls.2016.00591

**Published:** 2016-05-12

**Authors:** Geetika Sirhindi, Mudaser Ahmad Mir, Elsayed Fathi Abd-Allah, Parvaiz Ahmad, Salih Gucel

**Affiliations:** ^1^Department of Botany, Punjabi UniversityPatiala, India; ^2^Plant Production Department, College of Food and Agricultural Sciences, King Saud UniversityRiyadh, Saudi Arabia; ^3^Department of Botany and Microbiology, College of Science, King Saud UniversityRiyadh, Saudi Arabia; ^4^Department of Botany, S. P. CollegeSrinagar, India; ^5^Centre for Environmental Research, Near East UniversityNicosia, Cyprus

**Keywords:** antioxidants, growth, jasmonic acid, lipid peroxidation, nickel stress, osmolytes, reactive oxygen species, soybean

## Abstract

In present study, we evaluated the effects of Jasmonic acid (JA) on physio-biochemical attributes, antioxidant enzyme activity, and gene expression in soybean (*Glycine max* L.) plants subjected to nickel (Ni) stress. Ni stress decreases the shoot and root length and chlorophyll content by 37.23, 38.31, and 39.21%, respectively, over the control. However, application of JA was found to improve the chlorophyll content and length of shoot and root of Ni-fed seedlings. Plants supplemented with JA restores the chlorophyll fluorescence, which was disturbed by Ni stress. The present study demonstrated increase in proline, glycinebetaine, total protein, and total soluble sugar (TSS) by 33.09, 51.26, 22.58, and 49.15%, respectively, under Ni toxicity over the control. Addition of JA to Ni stressed plants further enhanced the above parameters. Ni stress increases hydrogen peroxide (H_2_O_2_) by 68.49%, lipid peroxidation (MDA) by 50.57% and NADPH oxidase by 50.92% over the control. Supplementation of JA minimizes the accumulation of H_2_O_2_, MDA, and NADPH oxidase, which helps in stabilization of biomolecules. The activities of superoxide dismutase (SOD), peroxidase (POD), catalase (CAT), and ascorbate peroxidase (APX) increases by 40.04, 28.22, 48.53, and 56.79%, respectively, over the control in Ni treated seedlings and further enhancement in the antioxidant activity was observed by the application of JA. Ni treated soybean seedlings showed increase in expression of *Fe-SOD* by 77.62, *CAT* by 15.25, *POD* by 58.33, and *APX* by 80.58% over the control. Nevertheless, application of JA further enhanced the expression of the above genes in the present study. Our results signified that Ni stress caused negative impacts on soybean seedlings, but, co-application of JA facilitate the seedlings to combat the detrimental effects of Ni through enhanced osmolytes, activity of antioxidant enzymes and gene expression.

## Introduction

Plants being sessile experience variety of biotic and abiotic stresses. Among the abiotic stresses, metal toxicity is most prevalent factor for polluting the soil and water bodies globally (Ahmad et al., 2015b). Some metals are essential for the normal functioning of plant cell, however, few are noxious and hampers plant growth and development (Ahmad et al., 2012b, 2015b). Nickel (Ni) is one of the essential metal elements required in small amounts by the plants. At high concentrations, Ni is considered to be highly toxic element as it enters the food chain easily and causes carcinogenesis (Kasprzak et al., 2003). Anthropogenic release of Ni from electroplating and steel industries constitutes main source of Ni pollution (Salt et al., 2000). Ni is required by plants for their normal metabolic activities including ureolysis, hydrogen metabolism, methane biogenesis acetogenesis (Mulrooney and Hausinger, 2003) and activates several other enzymes (Andreeva et al., 2001). However, excess of metals in agricultural soils cause osmotic and ionic stress to many crop plants (Ahmad et al., 2015b). Ni toxicity hampers seed germination and plant growth in terms of shoot and root length (Yusuf et al., 2011). The root growth inhibition may be due to the obstruction in mitotic activity (Gajewska et al., 2006).

Nickel stress decreases the pigment content, which leads to leaf chlorosis and necrosis (Gajewska et al., 2006; Seregin and Kozhevnikova, 2006; Ahmad et al., 2007). Ni is reported to inhibit electron transport chain, inactivates photosystem I (PSI) and II (PSII) (Tripathy et al., 1983) and blocks chlorophyll synthesis (Prasad and Prasad, 1987). The chlorophyll fluorescence parameters like F_0_ (initial fluorescence), F_m_ (maximum fluorescence), F*_v_* (variable fluorescence), F_v_/F_0_ (maximum pry. yield of photochemistry of photosystem PSII) are good indicators of abiotic stress (Javed et al., 2011). Low concentration of Ni increases the protein content (Singh and Pandey, 2011), however, at high concentrations it showed decline. The compatible solutes, proline and glycine betaine protects the cell from negative effects of metal stress due to their multiple functions (Sakamoto and Murata, 2002; Ahmad et al., 2015a,b). Proline content showed exponential increase with increasing concentrations of Ni (Singh and Pandey, 2011).

Ni toxicity also leads to generation of reactive oxygen species (ROS), e.g, H_2_O_2_, OH^⋅^ and O_2_^-^ that are highly reactive and cause oxidative damage to biomolecules (Ahmad, 2013; Ahmad et al., 2015a,b). Ni induced accumulation of H_2_O_2_ in leaves and roots of *Triticum aestivum* is reported by Hao et al. (2006) and Gajewska and Sklodowska (2007). Increased H_2_O_2_ enhanced the lipid peroxidation and is also reported by Ahmad et al. (2015a,b). Ni-stress have been reported to hamper fixation of CO_2_ and photosynthesis, suppresses electron transport and disrupts chloroplast (Gajewska et al., 2006; Ahmad et al., 2007).

Plants combat oxidative stress with low molecular weight compatible solutes along with an array of non-enzymatic and enzymatic antioxidants like superoxide dismutase (SOD), peroxidase (POD), ascorbate peroxidase (APX), catalase (CAT) and ascorbic acid (AsA). These antioxidants showed up or down regulation under stress and confer tolerance to plants (Ahmad et al., 2015a,b). Stress induced activity of enzymatic antioxidants was also reported in *Brassica juncea* (Ahmad et al., 2015a,b), maize (Bai et al., 2011), and chickpea (Ahmad et al., 2016). Metal stress has been reported to enhance the expressions of SOD genes (*Fe-SOD*, *Cu/Zn-SOD*) in soybean seedlings (Hossain et al., 2012). Enhanced *POD*, *APX* gene expression has also been reported in perennial ryegrass in response to metal stress (Li et al., 2012).

Jasmonic acid (JA) is the earnest candidate of plant growth regulator (PGR) family occurring ubiquitously in higher plants with diverse roles in plant growth and development (Wasternack and Hause, 2013; Wasternack, 2014). JA is also having a leading role as signaling molecule in plants under different environmental stresses (Wasternack and Hause, 2013; Wasternack, 2014; Kamal and Komatsu, 2016). JA applied externally in small amounts enhanced plant tolerance against abiotic stresses (Alam et al., 2014; Chen et al., 2014), plant growth and gene expression (Creelman and Mullet, 1995; Cheong and Choi, 2003).

Soybean (*Glycine max* L.) belongs to Fabaceae family and is cultivated for edible beans. Soybean contains important proteins (40%) and is also rich in amino acids for human and animal nutrition. It is also a major source of vegetable oil and the yield is decreasing due to various abiotic stresses including Ni toxicity (Abdel Latef et al., 2016). The aim of the work was to (i) study the impact of Ni toxicity on soybean seedlings and the mitigating role of JA, because JA is less studied to impart the metal stress tolerance and (ii) bridge the gap between physiological, metabolic and molecular aspects of Ni stress. So the present study was undertaken to investigate the effect of Ni and JA individually as well as in combination on growth, biochemical aspects, Chl fluorescence, and antioxidant defense system in *Glycine max.*

## Materials and Methods

### Collection of Seeds and Experimental Setup

Viable and certified seeds of *Glycine max* L. cv. SL-525 were surface sterlized in 5% sodium hypochlorite (NaOCl) solution for 10 min. After this, seed priming was done with 1 nM concentration of JA (Sigma chemicals, USA), for 8 h. JA treated and untreated seeds were grown in autoclaved petri dishes lined with Whatman filter paper placed in growth chamber under average day/night temperature of 25°C/16°C and with 80% relative humidity. The 4-day old germinated seedlings were shifted to plastic trays (10 plants per tray) containing peat, perlite and sand (1:1:1,v/v/v) supplemented with 2 mM Ni solution (NiCl_2_⋅6H_2_O, Sigma chemicals, USA). Control plants were fed with distilled water only. Each treatment is mean of five replications laid in randomized block design and each replicate includes five plants. The plant samples were collected for analysis after 15 days after treatment (DAT).

### Growth and Biomass Yield

The length of shoot and root are measured manually by scale. For the dry weight (DW) the plant samples (shoot, root, and leaves) were dried at 70^o^C in oven for 48 h and then weighted.

### Estimation of Total Chlorophyll (Total Chl)

Total Chl content in leaves were estimated by the method of Lichtenthaler (1987). The optical density (OD) was taken at 645, 663 nm by spectrophotometer (Beckman 640 D, USA) against 80% acetone used as blank.

### Analysis of Photosystem (PS) II Quantum Yield in Terms of F_v_/F_m_, F_0_/F_m_, qP, and NPQ

PS II quantum yield was determined with an imaging pulse amplitude modulated fluorometer (IMAG-MAXI; Heinz Walz) and calculated by the method described by White and Critchley (1999).

### Estimation of Proline and Glycine Betaine (GB) Content

Proline concentration was determined by the method previously described by Bates et al. (1973). Absorbance was determined spectrophotometrically at 520 nm (Beckman 640 D, USA) using toluene as blank.

GB content was determined according to the method of Grieve and Grattan (1983). The OD was taken at 365 nm by spectrophotometer (Beckman 640 D, USA). For the control GB (50–200 mg ml^-1^) was dissolved in 1N H_2_SO_4_.

### Estimation of Total Protein and Soluble Sugars

For the total protein content the method of Lowry et al. (1951) was employed. The OD was recorded at 595 nm by spectrophotometer (Beckman 640 D, USA) with bovine serum albumin as control.

The method of Dey (1990) was used for the estimation of total soluble sugars (TSSs). The absorbance was taken at 485 nm using a spectrophotometer (Beckman 640 D, USA).

### Measurement of Hydrogen Peroxide (H_2_O_2_) and Lipid Peroxidation (MDA)

For the estimation of H_2_O_2_ content, the procedure of Velikova et al. (2000) was followed. H_2_O_2_ content was calculated by using a standard curve with known concentrations and expressed as μM g^-1^ FW.

Lipid peroxidation [production of malondialdehyde (MDA)] was analyzed by the procedure previously described by Heath and Packer (1968). The absorbance was measured at 600 nm. Thiobarbituric acid (TBA) (1%) in 20% trichloroaceticacid (TCA) was used as blank.

### Estimation of NADPH Oxidase Activity

The NADPH oxidase activity was estimated by the method of Larsson et al. (1987). The plasma membrane was set apart from the cells with two phase aqueous polymer position system.

### Enzyme Assays

Fresh plant material (1g) was homogenized in 100 mM Tris-HCl (pH 7.5) in presence of DTT (Dithiothreitol, 5 mM), MgCl_2_ 10 mM, Ethylenediaminetetraacetic acid (EDTA, 1 mM), magnesium acetate 5 mM, Polyvinylpyrolidone (PVP-40 1.5%), phenylmethanesulfonyl fluoride (PMSF 1 mM) and aproptinin 1 μgmL^-1^. After the filtration, the homogenate was centrifuged at 10,000 rpm for 15 min. The supernatant collected after centrifugation served as enzyme source. For the analysis of APX activity, tissues were separately homogenized with 2 mM AsA. All experiments were performed at 4°C.

Activity of SOD was estimated according to Kono (1978) following the photo reduction of nitroblue tetrazolium (NBT). The absorbance was recorded spectrophotometerically (Beckman 640 D, USA) at 540 nm. SOD unit is the quantity of enzyme that hamper 50% photoreduction of NBT and is expressed as EU mg^-1^ protein.

The activity of POD was estimated according to the method proposed by Putter and Becker (1974). The rate of production of oxidized guaiacol was estimated spectrophotometerically (Beckman 640 D, USA) at 436 nm. The activity of POD was expressed as EU mg^-1^ protein.

Catalase activity was estimated by the method of Aebi (1984). The OD was taken spectrophotometerically (Beckman 640 D, USA) at 240 nm and the activity was expressed as EU mg^-1^ protein.

For the determination of APX activity, the procedure of Nakano and Asada (1981) was used. The OD was recorded at 265 nm by spectrophotometer (Beckman 640 D, USA) and the activity was expressed as EU mg^-l^ protein.

### Estimation of Ascorbic Acid

The method of Foyer et al. (1983) was employed for the estimation of AsA. The absorbance was recorded at 265 nm by spectrophotometer (Beckman 640 D, USA).

### Analysis of Gene Expression

Soybean plants treated with Ni and JA were further analyzed by real time polymerase chain reaction (RT-PCR). The extraction of total RNA from the leaves were carried out by using Trizol reagent (Promega). The RNA concentration and purity were determined spectrophotometrically at 260 and 280 nm. The first-strand cDNA was synthesized from 5 μg RNA template with GoScript^TM^ Reverse Transcription System (Promega) according to the manufacturer’s protocols with oligo (dT) 18 as a primer. cDNA was amplified by PCR using the primers (**Table 1**).

**Table 1:** Sequence of forward and reverse primers used in gene expression analysis.

Gene	Forward primer	Reverse primer
*FeSOD*	ATCTTAGTTATGGTTCTCTTTGT	ATGGTGTAGAGCCTTTTCATAT
*CAT*	AGCATCTCACCTGAACTTGAA	AGGTGAGAGGTTTGTGGCC
*POD*	TTGAAATAAAC CAAAGGAGTAGT	AATAATTATTTGAATCTCTTTAAGG
*APX*	CGTGACGATGATTGGGAAGT	TGATAGTGATCTTTCGGACCT
*SAc1*	AAGTGCTTCTAAATTGTTTGGTT	TGACAATGACATTGCAGAGAAT


To standardize the results, the relative abundance of β-actin (AB047313) was also determined, which was defined as 100 relative expression units (REU) and used as the internal standard.

### Statistical Analysis

The statistical analysis was executed by one-way analysis of variance (ANOVA) and Duncan’s Multiple Range Test (DMRT). The values represent the mean ± SE (*n* = 5). *P* ≤ 0.05 differ significantly.

## Results

## Ja Enhance Germination, Growth and Total Chl Content under Ni Stress

The results pertaining to the impact of nickel and JA on germination and growth of *Glycine max* are presented in **Table 2.** The Ni toxicity decreases the germination percentage by 29.59% as compared to control. However, application of JA to Ni- treated plants showed less decrease of 8.16% in germination rate over the control. Ni toxicity resulted in decline of both shoot and root length in the present study. The shoot length decreases by 37.23% with Ni treatment, however, plants treated with Ni in presence of JA showed increase in shoot length by 30.74% over control plants (**Table 2**). Root length declines by 38.31% in Ni stressed plants relative to control. Co-application of JA enhanced the root length by 70.06% as compared to Ni treated plants alone. JA treated control plants showed enhanced effect on shoot and root length in comparison to control (**Table 2**). Dry weight (DW) decreases by 14.88% in Ni stressed plants relative to control. Ni in combination with JA showed the increase in DW by 11.47% over the plants treated with Ni only (**Table 2**). Total Chl content declined by 39.21% in plants treated with Ni as compared to control plants. However, supplementation of JA to Ni treated plants increased the total Chl content by 38.70% over the plants treated with Ni alone (**Table 2**).

**Table 2:** Effect of Ni (2 mM) and JA individually and in combination on germination, shoot length, root length, dry weight, total chlorophyll in soybean seedlings.

Treatments	Germination	Shoot Length (cm)	Root Length (cm)	Dry weight (mg)	Total Chl mgg^-1^FW
Control	98 ± 0.061^a^	14.02 ± 0.14^b^	7.96 ± 0.29	78.33 ± 1.21^b^	0.51 ± 0.02^a^
JA	96 ± 0.062^a^	15.33 ± 0.19^b^	8.11 ± 0.31^a^	82.00 ± 1.65^a^	0.52 ± 0.01^a^
Ni	69 ± 0.060^c^	8.80 ± 0.32^c^	4.91 ± 0.12^c^	66.67 ± 1.03^d^	0.31 ± 0.005^c^
Ni + JA	90 ± 0.045^b^	18.33 ± 0.28^a^	8.35 ± 0.39^a^	74.32 ± 1.13^c^	0.43 ± 0.009^b^


*Data presented are the means ± SE (*n* = 5). Different letters next to the number indicate significant difference (*P* < 0.05).*

### JA Maintains Chlorophyll Fluorescence under Ni Stress

**FIGURE 1:**
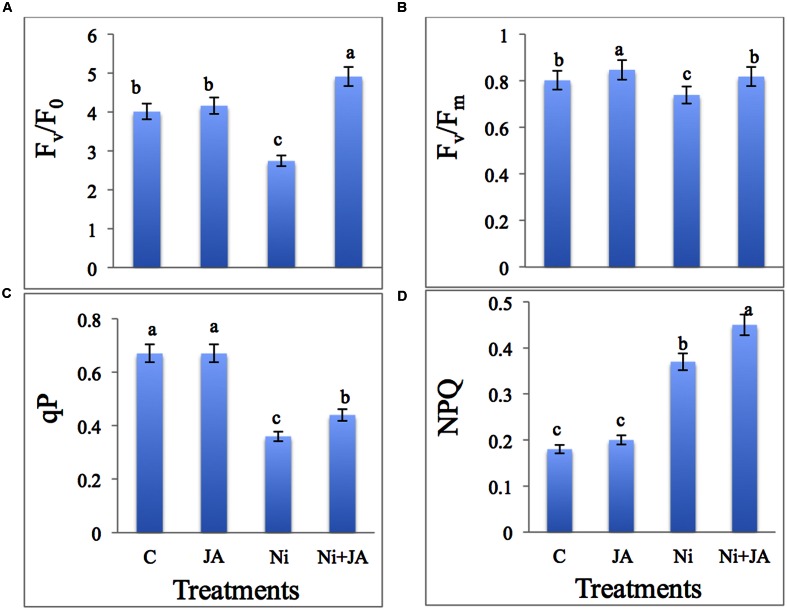
**Effect of Ni and JA individually and in combination on **(A)** F_v_/F_0_, **(B)** F_v_/F_m_, **(C)** qP, and **(D)** NPQ in soybean seedlings.** Different letters indicate significant difference between means at *p* ≤ 0.05 (DMRT). Values are means ± SE (*n* = 5).

Maximum primary yield (Fv/F_0_), Maximum quantum yield (Fv/Fm) of PS II photochemistry and photochemical quenching (qP) were markedly decreased by 31.67, 7.85, and 46.26%, respectively, under Ni stress in comparison to control plants. JA in combination with Ni showed significant increase by 79.19 in Fv/F0, 10.69 in Fv/Fm, and 22.22% in qP over the plants treated with Ni alone (**Figures 1A–C**). The results indicated that Fv/F_0_, Fv/Fm, and qP were higher in JA treated soybean plants. Non-photochemical quenching (NPQ) of soybean was increased significantly by 105.55% in Ni treated plants over the control. However, Ni treated plants supplemented with JA showed further increase by 21.62% as compared to Ni treated plants alone (**Figure 1D**).

### JA Improves Proline and Glycine Betaine Content under Ni Stress

Ni stress enhanced the proline content by 33.09% in soybean seedlings as compared to control. Co-application of JA to Ni fed plants showed further increase in proline content by 111.66% relative to plants treated with Ni alone (**Table 3**).

**Table 3:** Effect of Ni (2 mM) and JA individually and in combination on proline, glycine betaine, Total soluble sugar, protein content, and NADPH oxidase activity in soybean seedlings.

Treatment	Proline μgg^-1^FW	Glycine betaine μmolg^-1^ FW	TSS μgg^-1^FW	Protein mgg^-1^FW	NADPH oxidase activity o_2_^2-^ μmol min/mg prot.
Control	12.27 ± 1.28^d^	1.16 ± 0.01^c^	3.02 ± 0.11^d^	4.87 ± 0.31^d^	19.70 ± 1.18^c^
JA	15.42 ± 1.38^c^	1.19 ± 0.02^c^	3.43 ± 0.30^c^	4.93 ± 0.40^c^	19.53 ± 1.12^d^
Ni	18.34 ± 1.95^b^	2.38 ± 0.05^b^	5.94 ± 0.58^b^	3.77 ± 0.25^b^	40.14 ± 1.98^a^
Ni + JA	38.82 ± 2.55^a^	3.46 ± 0.08^a^	8.96 ± 0.95^a^	5.97 ± 0.75^a^	28.08 ± 1.41^b^


*Data presented are the means ± SE (*n* = 5). Different letters next to the number indicate significant difference (*P* < 0.05).*

GB increased by 51.26% in Ni treated plants over the control. Further increase in GB (45.37%) was recorded in Ni fed plants supplemented with JA compared to Ni treated plants alone (**Table 3**).

### JA Enhances Total Protein and Total Soluble Sugar under Ni Stress

The total protein content was decreased by 22.58% in Ni stressed plants over the control. However, addition of JA to Ni stressed plants showed elevation by 58.35% in protein content in comparison to plants treated with Ni only (**Table 3**).

The plants treated with Ni exhibited 49.15% increase in TSS over the control. However, co-application of Ni with JA recorded further increase by 50.84% in TSS as compared to plants treated with Ni alone (**Table 3**).

### JA Maintains H_2_O_2_ and MDA Level under Ni Stress

**FIGURE 2:**
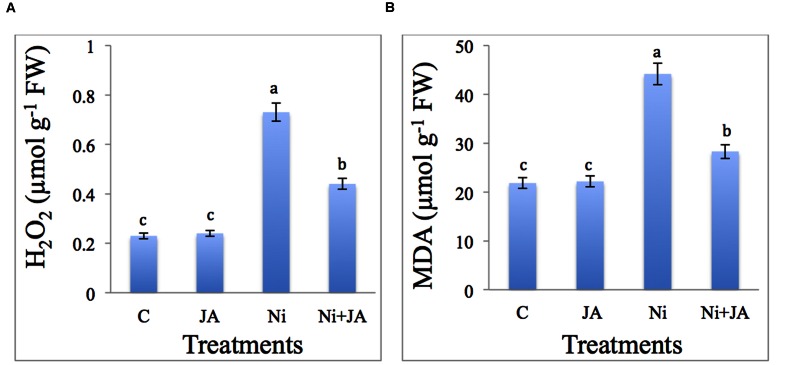
**Effect of Ni and JA individually and in combination on **(A)** H_2_O_2_ and **(B)** MDA content in soybean seedlings.** Different letters indicate significant difference between means at *p* ≤ 0.05 (DMRT). Values are means ± SE (*n* = 5).

The results related to the effect of Ni and JA individually and in combination on H_2_O_2_ and MDA content in soybean seedlings is presented in **Figures 2A,B.** The H_2_O_2_ content enhanced by 68.49% in Ni treated plants compared to control. However, supplementation of JA to Ni treated plants reduced the H_2_O_2_ content by 39.72% as compared to plants treated with Ni alone (**Figure 2A**).

Malondialdehyde content accumulated by 50.57% with Ni stress relative to control. Ni stressed plants supplemented with JA showed decline by 35.92% in MDA accumulation over the Ni treated plants alone (**Figure 2B**). Control plants supplemented with JA showed insignificant change in H_2_O_2_ and MDA content.

### JA Minimizes NADPH Oxidase under Ni Toxicity

Ni toxicity increased the NADPH oxidase by 50.92% in comparison to control. However, addition of JA to Ni stressed plants decreased the NADPH oxidase by 30.04% compared to Ni treated plants alone (**Table 3**).

### Effect of Ni and JA on Activity of Antioxidants

**FIGURE 3:**
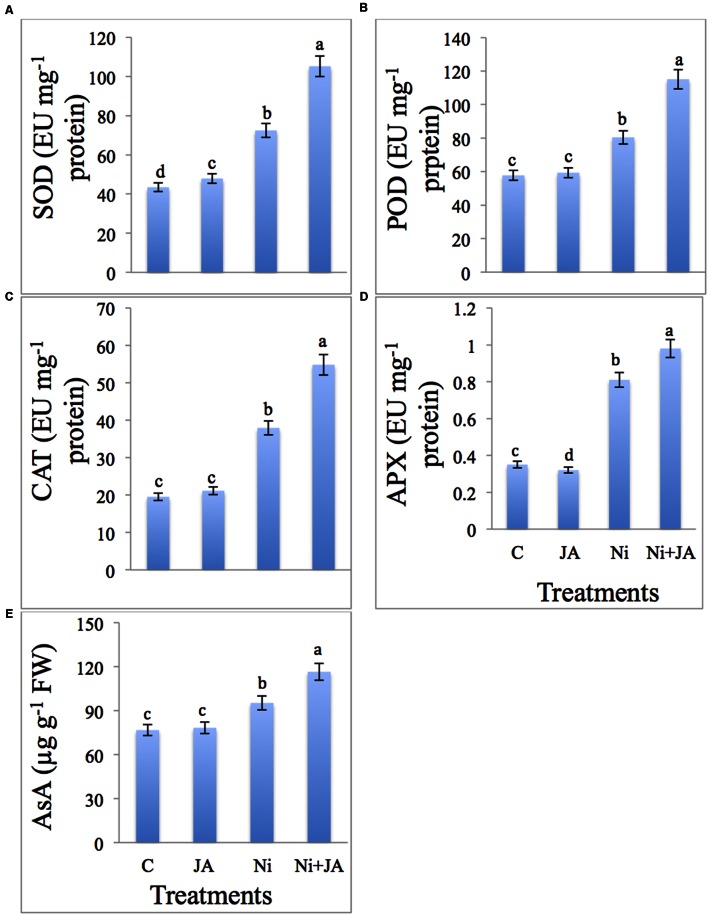
**Effect of Ni and JA individually and in combination on **(A)** SOD, **(B)** POD, **(C)** CAT, **(D)** APX, and **(E)** AsA in soybean seedlings.** Different letters indicate significant difference between means at *p* ≤ 0.05 (DMRT). Values are means ± SE (*n* = 5).

The results pertaining to the impact of Ni and JA on activities of enzymatic and non-enzymatic antioxidants are presented in **Figures 3A–E.** SOD activity was enhanced by 40.04% in Ni stressed plants; further increase by 45.17% was recorded by the application of JA to Ni stressed plants. POD, CAT, and APX were increased by 28.22, 48.53, and 56.79%, respectively, in Ni stressed plants over the control (**Figures 3B–D**). Application of JA to Ni stressed plants further enhanced the POD by 43.11, CAT by 44.47, and APX by 20.98% over the plants treated with Ni only. The AsA was increased by 19.42% in Ni treated plants over the control. Ni treated plants co-inoculated with JA showed further increase in AsA by 22.30% compared to plants treated with Ni alone (**Figure 3E**).

### Impact of Ni and JA on Antioxidant Gene Expression

**FIGURE 4:**
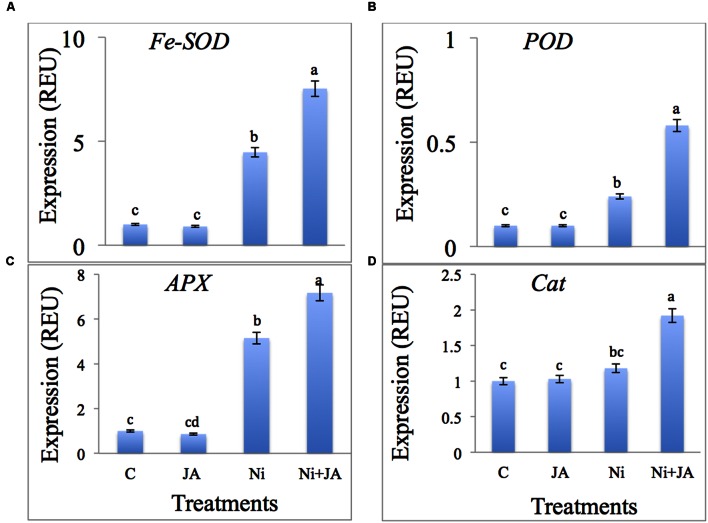
**Expression level of **(A)***Fe-SOD*, **(B)***POD*, **(C)***APX*, and **(D)***CAT* genes (REU relative expression unit) in soybean subjected to Ni and JA individually and in combination.** Different letters indicate significant difference between means at *p* ≤ 0.05 (DMRT). Values are means ± SE (*n* = 5).

The results related to the effect of Ni and JA on the expression levels of antioxidants is depicted in **Figures 4A–D.** Expression of *Fe-SOD* increased by 77.62% in Ni treated plants and 86.71% in plants treated with Ni in combination with JA over the control plants (**Figure 4A**). The expression of *POD*, *APX* and *CAT* increased by 58.33, 80.58, and 15.25%, respectively, in Ni stressed plants. However, JA supplementation to Ni treated plants further enhanced the expression levels of the above genes as compared to Ni treated plants alone (**Figures 4B–D**).

## Discussion

Ni is an essential micronutrient used by the plants for their normal growth and development. Ni has been reported to be an integral part of various biomolecules including metalloenzymes (Seregin and Kozhevnikova, 2006; Benoit et al., 2007). However, in excess quantity it proved to be toxic for the plant growth. The present study revealed decrease in germination percentage and growth by Ni toxicity (**Table 2**) and the results corroborate with the findings of Ahmad et al. (2007) in mung bean and Gajewska et al. (2006) in wheat. Ni treated plants showed poor root growth because of inhibition of mitotic activity, thus affects the overall growth of the plants (Gajewska et al., 2006). Application of JA improves the shoot and root length of the plant and may be due to less accumulation of Ni by the plant roots.

Ni toxicity drastically affects the pigment system in the present study (**Table 2**). Siddiqui et al. (2011) have also reported inhibition of chlorophyll content due to Ni toxicity in *T. aestivum*. Pigment concentration decreases with increase in concentration of Ni is also reported in *Pistia stratiotes* (Singh and Pandey, 2011), in *Brassica oleracea* (Pandey and Sharma, 2002), in *Vigna mungo* (Singh et al., 2012). Reduction in pigments due to Ni toxicity may be due to inhibition of α-aminolevulinic acid dehydratase (ALA-dehydratase) and proto chlorophyllide reductase involved in chlorophyll biosynthesis (Padmaja et al., 1990). Krupa et al. (1993) reported that photosynthetic electron intermediates (cytochrome b6f and b559) was affected by metal toxicity including Ni. Elevated levels of Ni enhance the H_2_O_2_ concentration, which resulted in chloroplast membrane peroxidation and may be one of the major reasons of decreased chlorophyll content under Ni stress (Dubey and Pandey, 2011). Keramat et al. (2010) have reported that application of JA improved shoot dry weight and total chlorophyll content in soybean under Cd stress. Yan et al. (2013) also reported that JA improved the chlorophyll content in *capsicum frutescens*. JA restored the chlorophyll content under cadmium stress is also reported in *Kandelia obovata* (Chen et al., 2014). Improved growth and chlorophyll content by external supplementation of JA might be due to: (i) JA hampers the uptake of Ni by roots, (ii) enhances mitotic activity in roots, (iii) JA is also responsible for the increase in CO_2_ fixation thus helps in enhancing photosynthetic rate (Fedina and Benderliev, 2000) and iv) JA helps in reduced accumulation of H_2_O_2_ and MDA content (Shan and Liang, 2010).

The F_v_/F_m_ representing the maximum quantum yield of PSII photochemistry is used as a stress indicator, while F_v_/F_o_ represents the active photosynthetic centers in the chloroplast of the plant (Israr et al., 2011). In this study, Fv/Fm ratio decreased with Ni treatment (**Figure 1B**). The change in ability of PSII under heavy metal stress was also reporterd by Burzyński and Klobus (2004) in *Cucumis sativus* L. The decline in F_v_/F_m_ and PSII efficiency demonstrated that Ni stress impede the photoactivation of PSII which may be due to destruction of antennae pigments and the limitation of Q_A_ (quinone) reoxidation by the decrease or partial block of electron transport from PSII to PSI (Mallick and Mohn, 2003). The exogenous application of JA increases the F_v_/F_m_ ratio, which indicates the plant is vigorous, and not suffering from photoinhibition. Ni treatment decreased F_v_/F_o_, reflects PSII donor side inhibition is linked with modification of the thylakoid membrane structure (Krupa and Baszynski, 1995). In numerous stress conditions increase in NPQ can often be accompanied by photoinactivation of PSII reaction centers, that leads to oxidative damage to the reaction centers and increase in F_0_ (Baker, 2008). In the present study NPQ increases accompanied with decrease in F_v_/F_m_ under Ni toxicity. The increase in NPQ might be due to inhibited photochemistry and is considered as additional mechanism to balance the excess absorbed light energy, thus prevents photoinhibition of PSII under Ni toxicity. JA restored the chlorophyll fluorescence is not elucidated yet, however, the reasons might be its role in (i) protecting the pigment systems form oxidative damage, (ii) maintaining the chlorophyll biosynthesis, as JA increases the Chl content in present study and (iii) increasing the capacity of plant cell to scavenge the ROS that destroys the membrane structures including chloroplast.

Ni toxicity increases proline content in the present study (**Table 3**) and the results coincide with the findings of Siddiqui et al. (2011) who also reported the enhancement in proline content in *T. aestivum* under Ni toxicity. Proline accumulation under heavy metal stress has been reported to be a potential indicator of stress tolerance (Ashraf and Foolad, 2007; Ahmad et al., 2015a,b). Proline assists in reconstruction of chlorophyll, activates Krebs cycle and constitutes an energy source (Ramon et al., 2003). Proline is also having a role in osmotic adjustment and stabilizes the macromolecules (Ahmad et al., 2012a,b). Proline has the ability to scavenge ROS and shields the cell from the oxidative damage (Ahmad et al., 2012a,b, 2015a,b). Glycine betaine is also increased with increase in Ni toxicity (**Table 3**) and is reported as an efficient compatible solute under abiotic stress (Munns, 2005). GB is having different roles like osmotic adjustment, maintain membrane integrity, stablizes PSII complex, safeguard Rubisco activity, and detoxification of noxious ions (Ashraf and Foolad, 2007). GB also maintains the protein structures from damage induced by abiotic stresses (Sakamoto and Murata, 2002). Under abiotic stress GB and proline have been reported to regulate gene expression by activating replication and transcription (Rajendrakumar et al., 1997). JA is reported to increase the proline content in *Cajanus cajan* under copper stress (Sharma et al., 2013) and might be due to stimulation of enzymes related to proline biosynthesis. Proline is having antioxidant property and might be induced by JA to protect the cell from oxidative burst. GB increases with JA treatments and the results corroborates with the findings of Gao et al. (2004). Exogenous application of JA significantly increases the betaine level in pear under drought stress and is due to up-regulation of BADH (betaine aldehyde dehydrogenase) expression (Gao et al., 2004).

Soluble proteins have been reported to decrease with the increase in heavy metal stress including Ni (Seregin and Kozhevnikova, 2006). The decrease in protein content in response to Ni and other heavy metal toxicity elevates protease activity, which resulted in degradation of proteins (Palma et al., 2002). Ni induced decrease in proteins may also be due to (i) Ni stress generates ROS that in turn cause damage to proteins (Gajewska et al., 2006) and (ii) heavy metals including Ni can bind to protein –SH groups and alter the protein structure and deplete the enzyme activity containing SH-groups (Seregin and Kozhevnikova, 2006). JA enhance the protein content in current study and the findings coincide with the reports of Sharma et al. (2013) on *C. cajan* under copper stress. JA has been reported to induce some proteins known as JISP (jasmonate induced stress protein) (Rakwal and Komatsu, 2001). It has been reported that various proteins produced during abiotic stress may be due to phytohormones like jasmonic acid (Thaler, 1999). JA has also been reported to increase the expression pattern of several proteins in peanuts under non-stress conditions (Kumari et al., 2006).

The increase in sugar content under Ni stress may be due to excessive resistance of photosynthetic organallae (Prokopiev, 1978) and less transport of starch from mesophyll cells. Elevated levels of heavy metals also disturb the carbon metabolism and are due to their negative impact on ribulos-bisphosphate carboxylase enzyme (Stiborová et al., 1987). Another reason of increased sugar content might be the starch degredation (Fischer and Holl, 1991). JA induced the accumulation of sugar content in *Pisum sativum* (El-Khallal, 2001), *Ipoema batata* (Ghoulam et al., 2002), *Brassica napus* (Kaur et al., 2013) under abiotic stress and is attributed to restoration of carbon metabolism and maximum export of starch from mesophyll cells. Accumulation of sugar may help the plants to absorb water from surroundings (Hajar et al., 1996).

H_2_O_2_ is a potent ROS and increases with increasing concentration of Ni (**Figures 2A,B**) and the findings coincides with the reports of Hao et al. (2006) in *Triticum* root. Gajewska and Sklodowska (2007) have also reported that Ni stress induced the accumulation of H_2_O_2_ in wheat leaves. Accumulation of H_2_O_2_ under heavy metal stress is also reported by many workers (Ahmad et al., 2015a,b). MDA is a product of lipid peroxidation and is commonly considered as oxidative stress indicator (Ahmad et al., 2012b, 2015a). Ni is reported to increase the hydrogen peroxide and LOX (Lipoxygenase is an oxidative enzyme) activity, which induced lipid peroxidation. Pandey and Rajeev (2010) also observed the increase in MDA content with increase in Ni concentration in eggplant. Furthermore, variety of metals increased the MDA content in *Bruguiera gymnorrhiza* and is thus regarded as the biomarker of metal stress (Zhang et al., 2007). Application of JA minimizes the production of H_2_O_2_ and other ROS, which directly affect the membrane lipids. The MDA decreases with external supply JA was also reported in *Kandelia obovata* under Cd stress (Chen et al., 2014). How JA is decreasing the H_2_O_2_ and MDA production is still unclear. However, it is assumed that JA enhanced the scavenging capacity of antioxidants that might lead to low production of H_2_O_2_ and ultimately decrease in lipid peroxidation. Another reason might be JA induced other endogenous phytohormones that could directly or indirectly impart tolerance to plants through low production of ROS (Kang et al., 2005).

Ni stress increases the PM NADPH in the present study (**Table 3**) and the results coincides with the findings of Hao et al. (2006) in *Triticum durum* roots. In plants PM NADPH oxidase is the key enzyme for the generation of ROS (Pei et al., 2000). It has been suggested that Ni stress enhanced the generation of ROS like H_2_O and O_2_^-^, that are responsible for the membrane lipid peroxidation, originate mainly from PM NADPH oxidase (Hao et al., 2006). Application of JA in the present study brings down the accumulation of PM NADPH oxidase (**Table 3**) and may be due to: (i) enhanced proline and GB synthesis, which has the antioxidant property and reduces the ROS generation and (ii) accumulation of enzymatic and non-enzymatic antioxidants that could also minimizes the production of ROS.

The increase in antioxidants activities in the present study (**Figures 3A–E**) coincides with the findings of Awasthi and Sinha (2013) in *Luffa cylindrical* under Ni stress. SOD, which is known as the first line of defense in plants, is observed to increase with Ni toxicity in eggplant (Pandey and Rajeev, 2010). Ni stress reported to increase the APX activity in different plants like, wheat (Gajewska and Skłodowska, 2008) and rice (Maheshwari and Dubey, 2009). Kumar et al. (2012) also reported the increase in guaiacol peroxidase (GPX), APX, SOD, and glutathione reductase (GR) activities in leaves and roots of barley seedlings against Ni stress. Piotrowska et al. (2009) also reported the enhanced activity of CAT, APX, NADH peroxidase, AsA in *Wolffia arrhiza* under lead toxicity. AsA in plants is very important phytoconstituent because of its antioxidant and cellular reductant property. It is also having multiple roles in growth and development of the plant especially under environmental stress. Antonious et al. (2011) have also reported increase in ascorbic content with different heavy metals including Ni. AsA has been reported to be a free radical scavenger under environmental stress. Supplementation of JA increases the activity of antioxidants in present study (**Figures 3A–E**). Chen et al. (2014) also reported that JA enhanced the CAT and APX activities in *K. obovata* seedlings subjected to Cd stress. AsA a potent non-enzymatic antioxidant was also increased with JA in *K. obovata* seedlings (Chen et al., 2014). Wolucka et al. (2005) reported that application of JA stimulates the AsA synthesis *Arabidopsis* and tobacco plants. It was reported that JA induces AsA might be due to enhanced expression of two late methyl jasmonate-responsive genes responsible for the enzyme synthesis for AsA biosynthesis. How the exogenous JA regulates the antioxidative enzyme activity is still in infancy. However, the positive role of JA on soybean plants may be due to: activation of genes responsible for Ni tolerance, that helps in detoxification of ROS. The detoxification might be interaction with superoxides directly or by enhancing the antioxidant enzyme capacity of the cell (Hsu and Kao, 2004). JA acts as a signaling molecule and is responsible for the induction of H_2_O_2_ and ROS signaling (secondary messengers) that could express the defense related genes and may be the reason for Ni tolerance to the cell.

Heavy metals stress has an impact on antioxidants and much of the research is going on the quantitative analysis and very less reports are there on gene expression (Bernard et al., 2015). Literature on antioxidant gene expression in plants under Ni toxicity supplemented with JA is very limited. *B. juncea* under Cd stress showed enhanced expression in catalase 3 gene (*CAT3*) and is reported by Minglin et al. (2005). Rasool et al. (2013) also reported the enhanced gene expression of *SOD*, *APX*, and *CAT* in chickpea under NaCl stress. Qilin et al. (2009) reported a new peroxidase gene (*OvRCI*) from *Orychophragmus violaceus* and quantitative real time PCR analysis showed it as salt inducible gene. Abu-Romman and Shatnawi (2011) explore the expression of *SOD* genes through RT-PRC and found *HvSOD* (*Hordeum vulgare*) gene is associated with antioxidant property and can express under environmental stress. In *Arabidopsis thaliana*, application of JA up-regulated the expression of oxidative stress genes (Jung et al., 2007).

## Conclusion

Increase of Ni concentration in the soil is a major threat to plant growth and crop production. Osmotic and oxidative stress induced by Ni toxicity negatively affect pigment system, chlorophyll fluorescence, and expression of antioxidants in the present study. Application of JA mitigates the harmful effects of Ni toxicity through the modulation of compatible solutes, antioxidants, and expression of *SOD*, *POD*, *APX*, and *CAT* genes. Use of JA in conveying the uncultivated land affected with Ni under cultivation will be a sustainable approach to increase the crop production.

## Author Contributions

GS, MM, and PA designed the experiment. PA, MM, GS and EFAA have written the manuscript. SG has contributed in discussion apart from statistical analysis and formatting of the paper.

## Conflict of Interest Statement

The authors declare that the research was conducted in the absence of any commercial or financial relationships that could be construed as a potential conflict of interest.
